# Atypical skeletal involvement in patients with Erdheim–Chester disease: CT imaging findings

**DOI:** 10.1186/s13023-022-02185-0

**Published:** 2022-02-03

**Authors:** Zaizhu Zhang, Wei Yu, Wenmin Guan, Qiang Lin, Ali Guermazi

**Affiliations:** 1 Department of Radiology, Peking Union Medical College Hospital, Chinese Academy of Medical Science and Peking Union Medical College, No.1 Shuaifuyuan Wangfujing Dongcheng District, Beijing, 100730 China; 2 Department of Radiology, Beijing Friendship Hospital, Capital Medical University, Beijing, 100050 China; 3 Department of Radiology, Beijing Arion Cancer Center, Beijing, 100050 China; 4 Department of Radiology and Medicine, Boston University School of Medicine and Boston Medical Center, 820 Harrison Avenue, FGH Building, 3rd Floor, Boston, MA 02118 USA

**Keywords:** Erdheim–Chester disease, Atypical skeletal involvement, Computed tomography, Imaging findings

## Abstract

**Objectives:**

To review retrospectively atypical bone findings from computed tomographic (CT) imaging in patients with Erdheim–Chester disease.

**Methods:**

All 28 patients with Erdheim–Chester disease (13 men and 15 women; mean age, 45 years; range, 7–63 years) underwent chest-abdomen-pelvis CT. CT images were reviewed and analyzed for the various features of atypical bone lesions by two radiologists in consensus.

**Results:**

Twenty-one patients had atypical bone involvement. Radiologically, these atypical osseous lesions were categorized into three types: diffuse, nodular and patchy. Eleven (52%) of the 21 patients had spinal lesions, of which four (36%) had the diffuse type, eight (73%) had the nodular pattern, and six (55%) had the patchy pattern. Sixteen (76%) of the 21 patients had pelvic involvement, of which two (13%) were diffuse, nine (56%) were nodular and 11 were (69%) patchy. Ribs were involved in seven (33%) of the 21 patients, with the nodular pattern in one (14%) patient and the patchy type in six (86%) patients. Clavicle involvement was seen in nine (43%) of the 21 patients, of which the diffuse type was found in only one (11%) patient, the nodular type in six (67%) patients, the solitary patchy type in four (44%) patients. Sternum involvement was seen in 10 (48%) of the 21 patients and all were nodular.

**Conclusions:**

This series provides a detailed description of atypical bone involvement in Erdheim–Chester disease which on CT displays three major patterns. Understanding these patterns may help increase the accuracy of diagnosis of this disease.

## Background

Erdheim–Chester disease (ECD) is a rare, non-Langerhans histiocytosis characterized by the infiltration of tissues by foamy CD68^+^CD1a^−^ histiocytes [1–3]. It is a systemic and neoplastic disorder, first described by Jakob Erdheim and William Chester in 1930 [4]. ECD can affect almost all systems and organs, and is frequently multisystem, with bone the most commonly affected, and osteosclerosis in 95% of patients [5].

The rarity of ECD, coupled with its diverse presentations, can make the diagnosis extremely elusive and require integration of often descriptive pathology with clinical, and radiographic findings. Given that the clinical and radiologic presentations of extraosseous involvement in ECD are diverse and nonspecific, the distinctive imaging findings of its skeletal involvement provide an important clue to the accurate diagnosis of this condition. The typical skeletal findings include a predilection for the long tubular bones of the appendicular skeleton, some degree of symmetry, and diffuse osteosclerosis that appears predominantly in the meta-diaphysis [6, 7].

The typical imaging findings of long tubular bones in ECD have been systemically described [6], however, rare and atypical skeletal involvement, including spine, pelvis (ilium, ischium and pubis), ribs, clavicles, and sternum, has been described generally in case reports or mentioned lightly in small series [5, 8–17]. Thus, the literature lacks a systematic description of a substantial series of patients with ECD. Additionally, compared with radiographs and magnetic resonance imaging (MRI), computed tomography (CT) has the advantage of showing osteosclerosis, which is the most common imaging features for ECD, and observed in 80–95% of ECD patients [2, 3, 5, 6, 18]. Our study aimed to describe atypical skeletal involvement (spine, pelvis, ribs, clavicles, and sternum) in ECD by retrospectively reviewing CT images of the chest-abdomen-pelvis with abnormal findings in 28 patients with ECD.

## Methods

This study was designed as a retrospective review. Both institutional review board approval and informed patient consent were waived for retrospective analyses of the patients’ medical records and imaging data.

### Patients and criteria for diagnosis

We retrospectively collected data of 28 patients with bone lesions (13 men and 15 women; mean age, 45 years; range, 7–63 years) who received a diagnosis of ECD from our hospitals between January 2014 and July 2020. Bone involvement was asymptomatic in 20 patients. Seven patients had bone pain of the extremities, and one had back pain. The diagnosis of ECD was established based on widely accepted criteria for ECD [1], including typical histopathologic and radiologic findings. Typical histologic findings are infiltration of typically foamy or lipid-laden histiocytes with admixed or surrounding fibrosis with immunostaining positive for CD68 and negative for CD1a. Characteristic skeletal findings are bilateral symmetric osteosclerosis of the meta-diaphysis of the lower extremity bones on studies such as radiographs, CT, or MRI. All 28 patients underwent biopsy of at least one lesion that was reviewed and confirmed independently by two pathologists.

### Imaging examination

All 28 patients underwent CT of the chest-abdomen-pelvis, performed on a dual-energy CT scanner, Discovery CT750 (GE Healthcare) or Somatom Definition Flash (Siemens Healthineers). Skeletal involvement, including spine, pelvis, ribs, clavicles, and sternum, was delineated from the bone window (width, 2000 HU; level, 350 HU). Although our imaging center is equipped with scanners from different manufacturers, standardization (e.g., scan range, phases, slice thickness) is strictly implemented for the same examination to ensure that the images from different scanners are comparable and have similar image quality.

### Image analysis

The CT images were retrospectively reviewed in consensus by two radiologists. These lesion characteristics were analyzed: location (spine, pelvis, ribs, clavicles, and sternum), distribution (unilateral or bilateral; symmetric or asymmetric), range (diffuse or focal), shape (nodular or patchy), number (solitary or multiple), density changes on CT images (osteosclerosis or osteolysis), cortical bone thickening (absent or present), bone marrow cavity (normal or narrowed), and cortical bone–medullary cavity margin (normal or blurred), which were summarized in Table 1. Associated findings such as joint destructions, fractures and expansive changes were also noted.

**Table 1:** The image analysis of Erdheim–Chester Disease characteristics on CT images

Characteristics	Details
Location	Spine
	Pelvis
	Ribs
	Clavicles
	Sternum
Distribution	Unilateral
	Bilateral
	Symmetric
	Asymmetric
Range	Diffuse
	Focal
Shape	Nodular
	Patchy
Number	Solitary
	Multiple
Density changes	Osteosclerosis
	Osteolysis
Cortical bone thickening	Absent
	Present
Bone marrow cavity	Normal
	Narrowed
Cortical bone–medullary cavity margin	Normal
	Blurred
Joint destructions	Absent
	Present
Fractures	Absent
	Present
Expansive changes	Absent
	Present

## Results

Of the 28 patients with ECD, 21 (75%) had abnormal atypical skeletal imaging findings and seven had normal CT images. The 21 patients (mean age, 45 years; age range, 7–63 years) with abnormal examination results, which served as the basis for this study, were 10 males (mean age, 44 years; age range, 7–60 years) and 11 females (mean age, 45 years; age range, 24–63 years). The abnormal findings were summarized in Table 2.

**Table 2:** Imaging findings of atypical osseous lesions in 21 patients with Erdheim–Chester Disease

Findings	Spine	Pelvis	Ribs	Clavicles	Sternum
Axial skeleton involved	11 (52)	16 (76)	7 (33)	9 (43)	10 (48)
Distribution					
Unilateral	–	6 (38)	3 (43)	3 (33)	–
Bilateral	–	10 (63)	4 (57)	6 (67)	–
Symmetric	–	0 (0)	0 (0)	1 (17)	–
Asymmetric	–	10 (100)	4 (100)	5 (83)	–
Type					
Diffuse type	4 (36)	2 (13)	0 (0)	1 (11)	0 (0)
Homogeneous	0 (0)	0 (0)	0 (0)	0 (0)	0 (0)
Heterogeneous	4 (100)	2 (100)	0 (0)	1 (100)	0 (0)
Focal type					
Nodular pattern	8 (73)	9 (56)	1 (14)	6 (67)	10 (100)
Solitary	2 (25)	1 (11)	0 (0)	3 (50)	5 (50)
Multiple	6 (75)	8 (89)	1 (100)	3 (50)	5 (50)
Patchy pattern	6 (55)	11 (69)	6 (86)	4 (44)	0 (0)
Solitary	0 (0)	5 (45)	2 (33)	4 (100)	0 (0)
Multiple	6 (100)	6 (55)	4 (67)	0 (0)	0 (0)
Fracture	0 (0)	0 (0)	0 (0)	0 (0)	0 (0)
Expansion	0 (0)	0 (0)	4 (57)	0 (0)	0 (0)

**Fig. 1:**
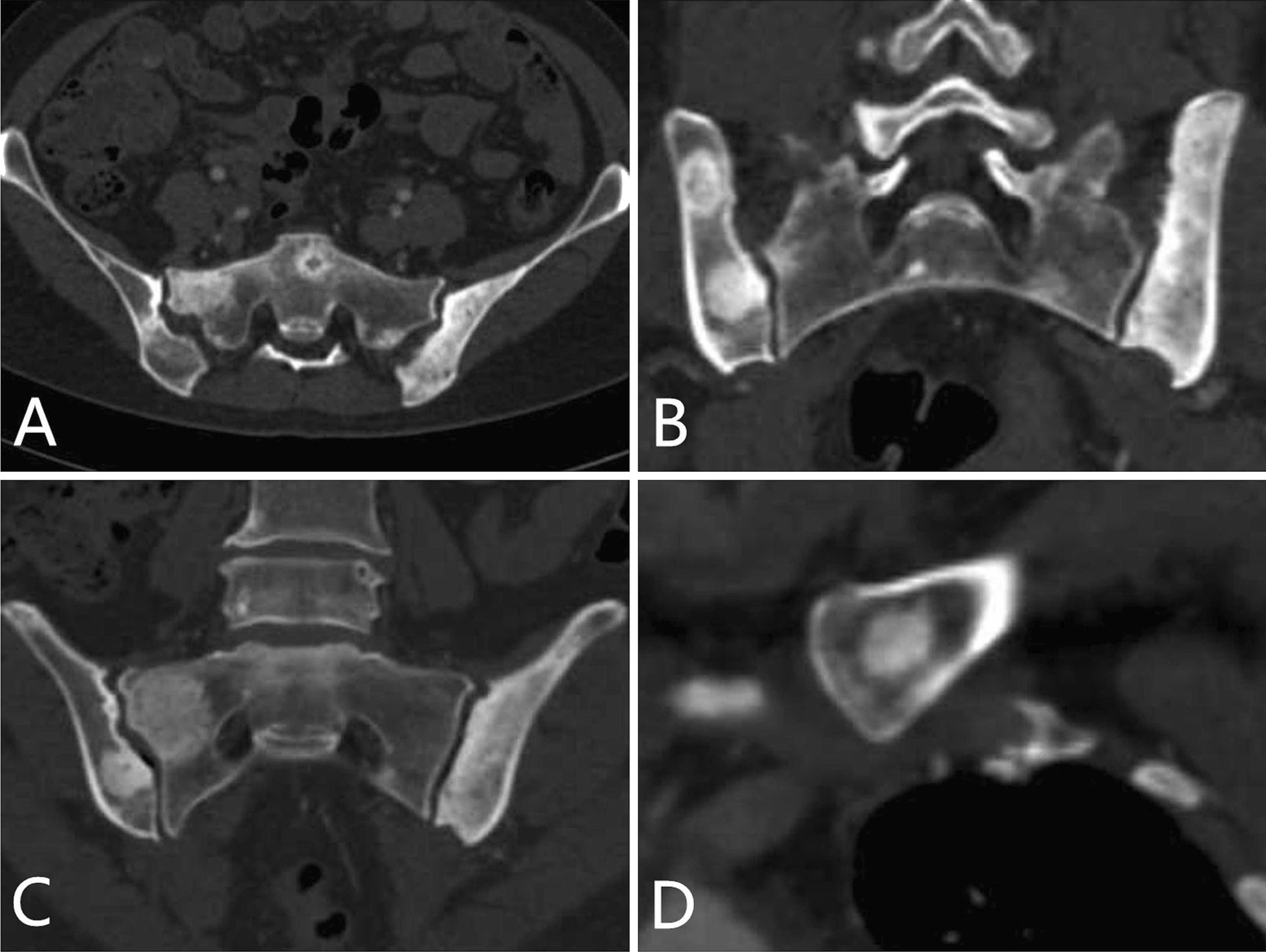
CT images in a 47-year-old woman with pelvis, spine, and clavicles involved. **A** Axial and **B**, **C** coronal reconstruction pelvic CT images show heterogeneous diffuse osteosclerosis filling the left iliac bone, as well as multiple focal osteosclerotic lesions with diverse appearance of nodular and patchy shape in the right iliac bone and the sacrum. **D** Coronal reconstruction chest CT image shows nodular osteosclerosis in the left proximal clavicle

**Fig. 2:**
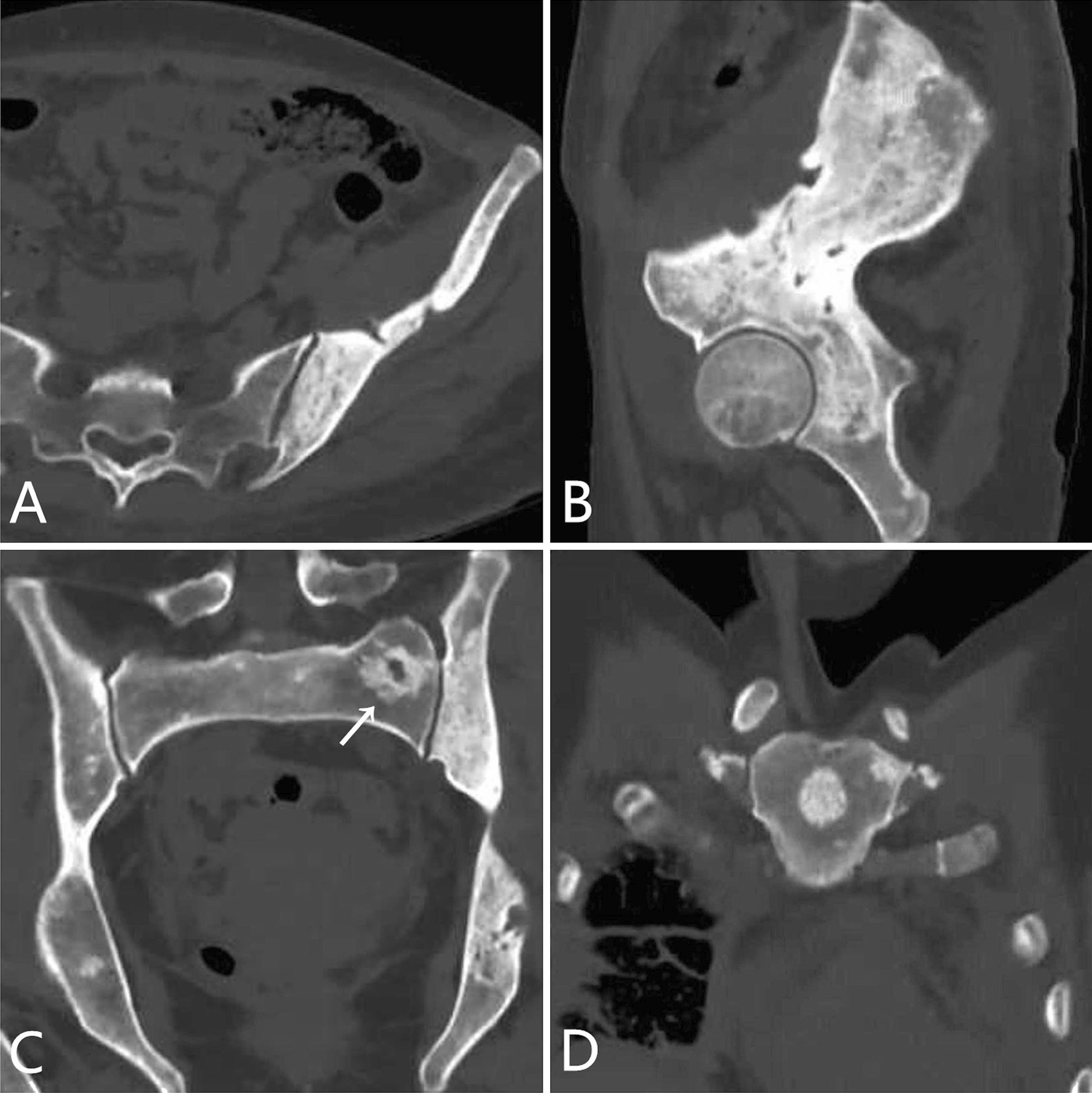
CT images in a 40-year-old woman with pelvis, spine, and sternum involved. **A** Axial and **B** sagittal reconstruction pelvic CT images show heterogeneous diffuse osteosclerosis filling the left iliac bone with multiple lucent foci and disseminated and multiple cortical osteolytic lesions. **C** Coronal reconstruction pelvic CT image shows a lytic lesion with sclerotic margin in the sacrum, looking like a ring (arrow). **D** Coronal reconstruction chest CT image shows solitary well-circumscribed, nodular osteosclerotic lesion in the sternum

**Fig. 3:**
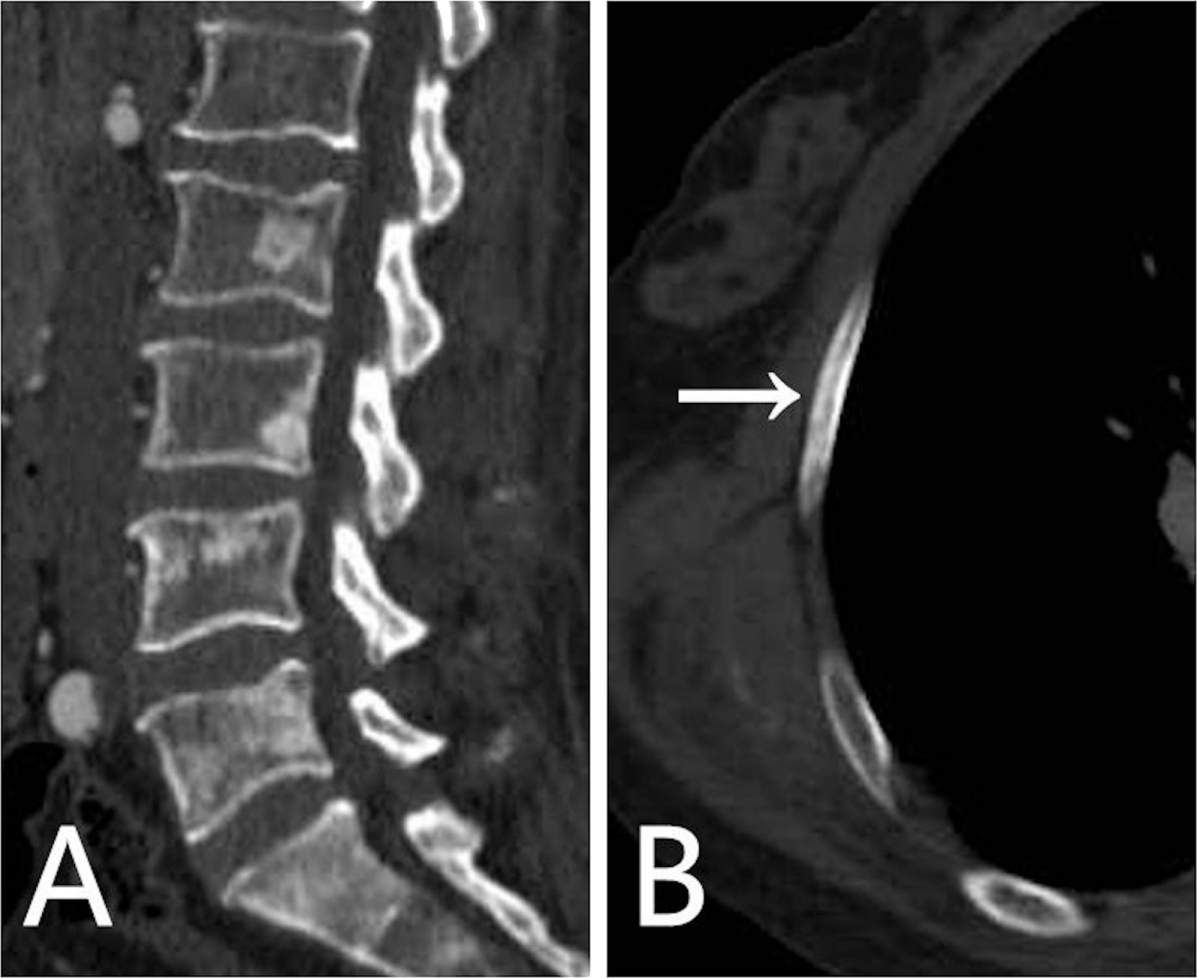
CT images in a 40-year-old woman with spine and ribs involved. **A** Sagittal reconstruction spine CT image shows heterogeneous diffuse osteosclerosis in the L-5 and S-1 vertebrae, and focal osteosclerosis in the L2-4 vertebrae. **B** Axial chest CT image shows patchy osteosclerotic lesion of the ninth right side rib without expansion (arrow)

**Fig. 4:**
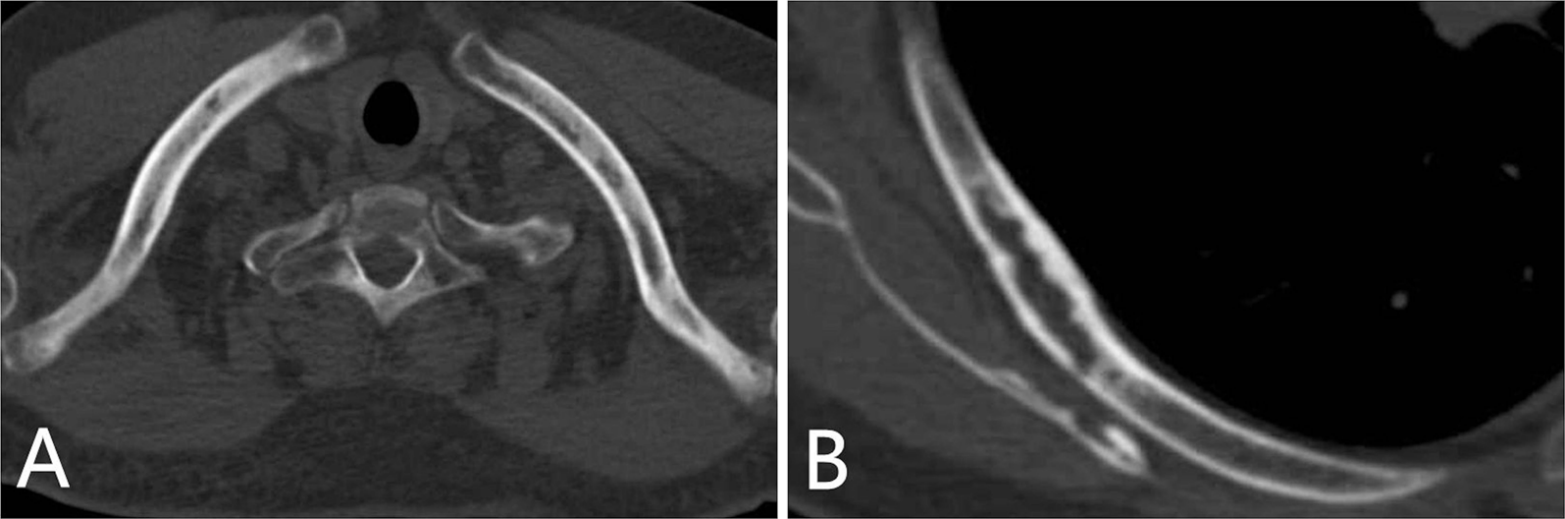
Chest CT images in a 45-year-old man with clavicles and ribs involved. **A** Axial chest CT image shows heterogeneous diffuse osteosclerosis filling both clavicles. **B **Axial chest CT image reveals a lytic lesion with a sclerotic margin and expansive change in the seventh right posterior rib

**Fig. 5:**
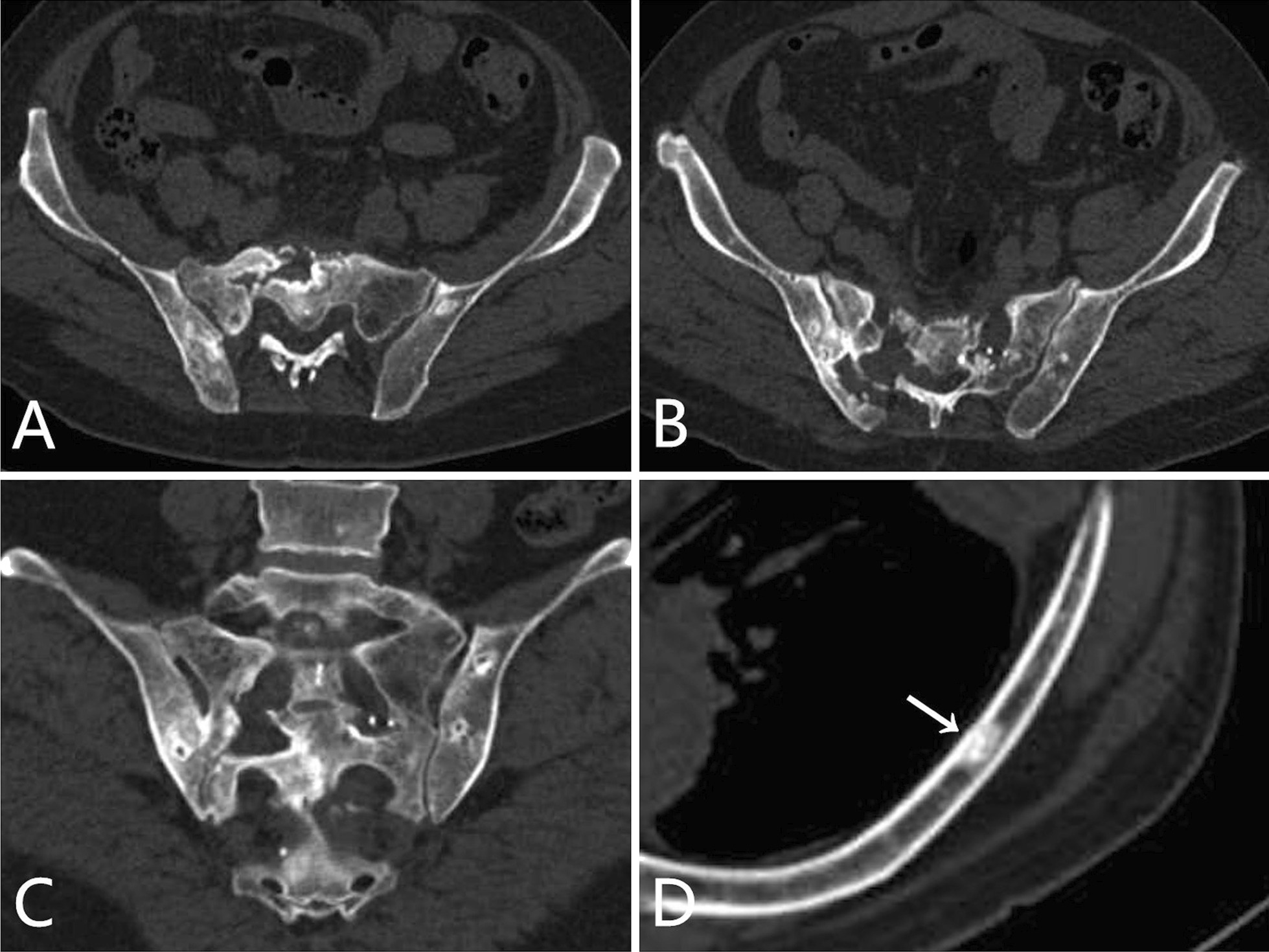
CT images in a 52-year-old man with pelvis, spine, and ribs involved. **A**–**B** axial pelvic CT images and **C** coronal reconstruction pelvic CT image show mixed osteolytic and osteosclerotic lesions in the right iliac bone and the sacrum with the destruction of the sacroiliac joint, as well as multiple lytic lesions with sclerotic margins in the right iliac bone. **D** Axial chest CT image demonstrates focal osteosclerotic lesion of the ninth left side rib without expansion (arrow)

The skeletal lesions in the 21 patients could be classified into two types: diffuse and focal. The diffuse type presented as heterogeneous diffuse osteosclerosis filling the bone with cortical thickening, a reduced corticomedullary cavity and blurring of corticomedullary differentiation on CT images. There were also multiple lucent foci less than 1 cm in diameter disseminated within the diffuse sclerotic lesions, creating a heterogeneous speckled appearance (Fig. 1A–C, 2A–C, 3A, 4A) or diffuse mixed osteosclerosis and osteolysis (Fig. 5A-C). The focal type was then divided by shape: nodular and patchy. The nodular pattern appeared as solitary or multiple, well-circumscribed, round or irregularly-shaped osteosclerotic lesions (Figs. 1A–D, 2D, 3A, 5D) or lytic lesions with sclerotic margins, looking like a ring (Figs. 1A, 2C, 5C). The patchy pattern exhibited solitary or multiple patchy areas of osteosclerosis with coarse trabeculae and cortical thickening (Fig. 3B) or mixed osteosclerosis and osteolysis (Fig. 4B).

### Spine

Spinal lesions were observed in 11 (52%) of the 21 patients. Eight (73%) of the 11 patients had the nodular pattern, of which six (75%) patients were multiple. The spinal lesions presented as heterogeneous diffuse type in four (36%) patients and multiple patchy patterns in six (55%) patients. In addition, no fractures or expansive changes were found in the spinal lesions of the 11 patients.

### Pelvis

Sixteen (76%) of the 21 patients had pelvic involvement, of which 10 (63%) were bilateral and asymmetric. Patchy patterns were observed in 11 (69%) and nodular patterns in nine (56%) of the 16 patients; multiple lesions were found in six (55%) patients with the patchy pattern and eight (89%) of those with the nodular pattern. The heterogeneous diffuse pattern appeared in only two (13%) patients. One patient showed destruction of the sacroiliac joint (Fig. 5B). No fractures or expansive changes were found in any of the pelvic lesions of the 16 patients.

### Ribs

Seven (33%) of the 21 patients showed costal involvement. Four (57%) had bilateral lesions, all of which were asymmetric. The patchy type was seen in six (86%) of the seven patients, four (67%) of which were multiple. Only one (14%) patient had the multiple nodular pattern. Expansive changes were observed in four (57%). No fractures were found in these lesions.

### Clavicles

Nine (43%) of the 21 patients had clavicular lesions. Bilateral lesions were observed in six (67%) of the nine patients, five (83%) of which were asymmetric. Lesions were solitary or multiple nodular in six (67%) of the nine patients, solitary patchy in four (44%) and heterogeneous diffuse in only one (11%) (Fig. 4A). No fractures or expansive changes were seen in these lesions.

### Sternum

Sternum involvement was seen in the 10 (48%) of the 21 patients and all exhibited the nodular pattern; with single lesion in five (50%) patients and multiple lesions in five (50%) others. No cortical destruction, fractures or expansive changes were deserved in these lesions.

## Discussion

We report the CT imaging findings of skeletal involvement in a series of 28 patients with ECD, with emphasis on some aspects that, to our knowledge, have not been systematically described before: radiological features of atypical bone involvement in the spine, pelvis, ribs, clavicles, and sternum. In contrast to a previous study which found that the axial skeleton is typically spared in ECD [6], 21 (75%) of 28 patients in our series had one or more affected sites in the axial skeleton. This can be explained by the systematic use of CT to evaluate ECD patients in our institution. In this study, most patients were middle-aged adults, which was consistent with other reports of ECD [2, 3, 18, 19]. The number of men and women, however, was similar, with no preponderance of men [2, 3, 18, 19]. While bone involvement is an almost constant finding in ECD, it is asymptomatic in at least 60% of patients [5]. In our study, only one patient had back pain associated with spinal involvement, showing how the lack of symptoms in these atypical bone sites can lead to misdiagnosis and highlighting the importance of recognizing the imaging features of atypical bone involvement.

Radiologically, bone lesions of ECD can be categorized into three types: diffuse, nodular, and patchy based on the range and shape. Atypical skeletal lesions generally appear as nodular and/or patchy, while the diffuse type is more common when the long bones are involved [6]. All of these lesions consisted of pure osteosclerosis, osteosclerosis with multiple lucent foci less than 1 cm in diameter, or mixed osteosclerosis and osteolysis, which may be consistent with their pathology [2, 3, 5, 20]. Of note, the diffuse lesions in our study all had a heterogeneous speckled appearance, which may be attributed to the high resolution of CT.

In our study, spinal lesions were observed in 52% of the 21 patients exhibiting nodular, patchy, diffuse or mixed patterns, with the multiple nodular type the most common, similar to the multifocal lesions reported by Klieger et al. [13]. The diffuse type was the most common in the literature [8, 10, 12, 14–16], possibly because the diffuse type is conspicuous and easy to detect. In line with the cases reported by Veyssier et al. [5], all the spinal lesions in our study were osteosclerotic. However, several cases with purely osteolytic lesions in the vertebra have been reported [9, 12]. We saw cortical destruction of sacral lesions with morphological abnormalities in two patients, which to our knowledge has not been reported before. Compression fractures of the spine have been reported [10], although none occurred in the 11 patients with spinal lesions in this study, possibly because the lesions were osteosclerotic.

The pelvis was the most frequently involved site, with lesions in 76% of the 21 patients. The distribution was unilateral or asymmetrically bilateral with the latter in the majority. Most pelvic lesions appeared as nodular and/or patchy, which was similar to the reports of Klieger et al. [13] and Zhu et al. [21]. The heterogenous diffuse osteosclerosis found in two patients does not seem to have been reported previously. In line with the report by Allmendinger et al. [8], of multiple cortical osteolytic lesions within the right iliac crest, five patients in our series had concurrent osteolytic destructions with one patient showing destruction of the sacroiliac joint.

In 33% of the 21 patients, the ribs were involved. Distribution was largely asymmetrically bilateral, and of the multiple patchy type with expansive changes, which was similar to the cases reported by Dalinka et al. [17]. There is little in the literature about clavicular and sternal involvement in ECD, and even less about imaging findings. Clavicular sclerosis and thickening in ECD were only touched on in the literature review by Walker et al. [22]. In our series, 43% of the 21 patients showed clavicular involvement, with a predilection for asymmetrically bilateral, solitary or multiple lesions, with the nodular pattern, and the 48% of patients with the sternum involved all presented as single or multiple nodular pattern.

Of note is that these atypical sites of osseous involvement in ECD are more typical of Langerhans cell histiocytosis (LCH). However, radiologically, in LCH these bone lesions often have the well-defined lytic “punched-out” appearance caused by asymmetric destruction of the inner and outer cortices [23]. Although few cases have reported the purely lytic appearance in ECD [9, 12], all the lesions in the current study had osteosclerosis, which can contribute to the differential diagnosis between ECD and LCH. Patients with LCH bone involvement usually present with bone pain, fractures, or cord compression, however, no fractures or cord compression were seen in our study. In addition, histopathologic analysis plays a key role in differentiating ECD from other types of histiocytosis. ECD differs from LCH in terms of the immunohistologic and microscopic characteristics of the histiocytes, which when associated with ECD are CD68/CD163 positive and CD1a negative and do not immunostain for S-100 protein and OKT6 [24].

Interestingly, new insights have been recently provided into the association of ECD with other histiocytic neoplasms, especially LCH. This entity called “mixed histiocytosis” was initially reported in several cases with both biopsy-proved osteolytic LCH lesions and diffuse sclerotic involvement of long bones characteristic of ECD which was later confirmed as ECD in a French series of 23 patients [2, 25–27]. In these cases, the ECD component was either diagnosed subsequently or concomitantly with LCH, but never preceded it; however, the phenotypes of patients with mixed histiocytosis were heterogeneous, but closer to isolated ECD than isolated LCH [3]. Owing to the co-occurrence of ECD with LCH in 15% of patients with ECD and the discovery of BRAF mutations and of other MAP kinase pathway alterations, LCH and ECD belong to the “L” group in the 2016 revision of the classification of histiocytosis [28]. In this regard, it is crucial for clinicians to be aware of the co-occurrence of these histiocytic neoplasms so that these atypical manifestations not only evoke the diagnosis of ECD, but also alert them toward consideration of another biopsy to confirm overlapping entities.

An early diagnosis is important to start disease-modulating therapy, which may improve prognosis and survival of patients, and is of significant importance to clinical studies recruiting for this disorder. If untreated, it can prove fatal, particularly in multisystem disease. In our study, we described diverse imaging findings of ECD with three patterns and several cases with isolated bone lesions of the spine and/or pelvis. Hence, it seems significant to have an intimate knowledge of these atypical skeletal imaging features in patients with ECD, which can provide an important clue to the accurate and early diagnosis, although the prognosis of the patient is determined by the extent and the distribution of the extra skeletal manifestations of the disease.

This study had several limitations. On the one hand, because of the rarity of ECD, the number of patients was relatively small; however, this was the largest sample of patients with atypical bone involvement to be published and contributed many rare imaging manifestations. On the other hand, even though we had histologic proof of ECD in each patient, we did not have direct histologic confirmation of each lesion assessed in this work; nonetheless, the lesions that were not biopsied shared similar imaging characteristics with biopsied lesions. Additionally, we only described the CT imaging findings of atypical bone involvements in ECD, and more imaging features need further research by other imaging modalities, particularly for MRI.

## Conclusions

We further described the appearance of atypical ECD bone lesions in a series of 28 patients and categorized the imaging findings into three different types: diffuse, nodular, and patchy pattern. Strong knowledge of atypical osseous CT manifestations in ECD should lead to accurate diagnosis as well as to a better understanding of the overall effects of the disease on the skeleton.

## Data Availability

Not applicable.
